# Supra­molecular patterns and Hirshfeld surface analysis in the crystal structure of bis­(2-amino-4-meth­oxy-6-methyl­pyrimidinium) isophthalate

**DOI:** 10.1107/S2056989017013950

**Published:** 2017-09-29

**Authors:** Muthaiah Jeevaraj, Palaniyappan Sivajeyanthi, Bellarmin Edison, Kaliyaperumal Thanigaimani, Kasthuri Balasubramani, Ibrahim Abdul Razak

**Affiliations:** a Department of Chemistry, Government Arts College (Autonomous), Thanthonimalai, Karur 639 005, Tamil Nadu, India; b Department of Chemistry, Government Arts College, Tiruchirappalli 620 022, Tamil Nadu, India; c School of Physics, Universiti Sains Malaysia, 11800 USM, Penang, Malaysia

**Keywords:** crystal structure, 2-amino­pyrimidines, isophthalates, hydrogen bonding, Hirshfeld surface analysis

## Abstract

In the title mol­ecular salt, 2C_6_H_10_N_3_O^+^·C_8_H_4_O_4_^2−^, the N atom of each of the two 2-amino-4-meth­oxy-6-methyl­pyrimidine mol­ecules lying between the amine and methyl groups has been protonated. The dihedral angles between the pyrimidine rings of the cations and the benzene ring of the succinate dianion are 5.04 (8) and 7.95 (8)°. Each of the cations is linked to the anion through a pair of N—H⋯O(carboxyl­ate) hydrogen bonds, forming cyclic *R*_2_^2^(8) ring motifs which are then linked through inversion-related N—H⋯O hydrogen bonds, giving a central *R*_2_^4^(8) motif. Peripheral amine N—H⋯O hydrogen-bonding inter­actions on either side of the succinate anion, also through centrosymmetric *R*_2_^2^(8) extensions, form one-dimensional ribbons extending along [211]. The crystal structure also features π–π stacking inter­actions between the aromatic rings of the pyrimidine cations [minimum ring centroid separation = 3.6337 (9) Å]. The inter­molecular inter­actions were also investigated using Hirshfeld surface studies and two-dimensional fingerprint images.

## Abstract 2

The structure of the substituted 2-amino­pyrimidine salt of isophthalic acid comprises an almost planar unit in which the two pyrimidinium cations are cyclically hydrogen-bonded to the succinate dianion and further extended through hydrogen bonds into a one-dimensional supra­molecular structure.

## Chemical context   

Pyrimidine and amino­pyrimidine derivatives have useful applications in many fields, for example as pesticides and pharmaceutical agents (Condon *et al.*, 1993 ▸), while imazosulfuron, ethirmol and mepanipyrim have been commercialized as agrochemicals (Maeno *et al.*, 1990 ▸). Pyrimidine derivatives have also been developed as anti­viral agents, such as AZT, which is the most widely used anti-AIDS drug (Gilchrist, 1997 ▸). Hydrogen bonding plays a vital role in mol­ecular recognition. It is significant to know the types of hydrogen bonds present to design new materials with highly specific features. Supra­molecular chemistry plays a pivotal role in many biological systems and is involved in artificial systems. It refers to the specific relation between two or more mol­ecules through non-covalent inter­actions such as hydrogen bonding, hydro­phobic forces, van der Waals forces and π–π inter­actions. The origin of supra­molecular architectures is correlated to the positions and properties of the active groups in mol­ecules (Desiraju, 1989 ▸; Steiner, 2002 ▸). As part of our recent studies in this field, the synthesis, crystal structure and Hirshfeld surface analysis of the title salt have been undertaken and are presented herein.
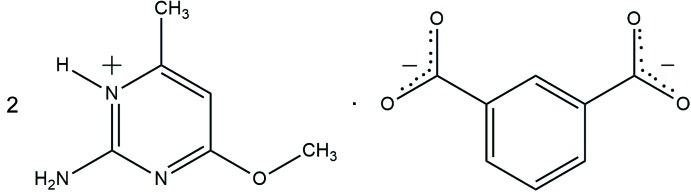


## Structural commentary   

**Figure 1:**
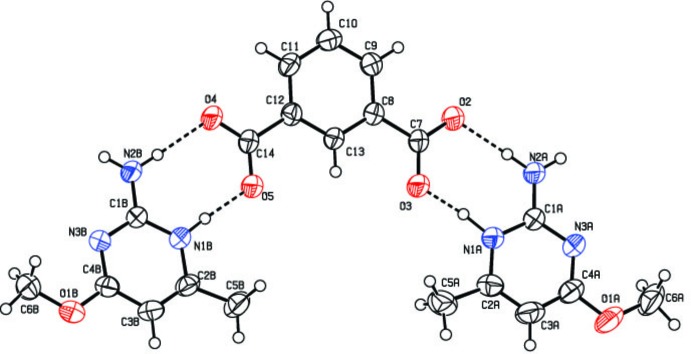
The atom numbering for the two cations and the dianion in the asymmetric unit of the title salt, with probability displacement ellipsoids drawn at the 50% probability level. Hydrogen bonds (Table 1 ▸) are shown as dashed lines.

The asymmetric unit of the title salt comprises two 2-amino-4-meth­oxy-6-methyl­pyrimidinium cations (*A* and *B*) and an isophthalate dianion (Fig. 1 ▸). The cations and the anion are essentially planar with the dihedral angles between the pyrimidine rings of cation *A* and cation *B* and that of the benzene ring of the succinate dianion of 5.04 (8) and 7.96 (8)°, respectively. The pyrimidinium cations are protonated at N1*A* and N1*B*, which are present between the amine and methyl groups. The protonation is reflected in an enhancement in bond angles at N1*A*/N1*B* [C1*A*—N1*A*—C2*A* = 120.76 (13)°; C1*B*—N1*B*—C2*B* = 120.99 (14)°], when compared with those at the unprotonated atom N3*A*/N3*B* [C1*A*—N3*A*—C4*A* = 116.01 (14)°; C1*B*—N3*B*—C4*B* = 116.45 (13)°]. The corres­ponding angle in neutral 2-amino-4-meth­oxy-6-methyl­pyrimidine (Glidewell *et al.*, 2003 ▸) is 116.01 (18)°. The bond lengths and angles are normal for the carboxyl­ate groups of the isophthalate anion (Allen *et al.*, 1987 ▸).

## Supra­molecular features   

**Figure 2:**
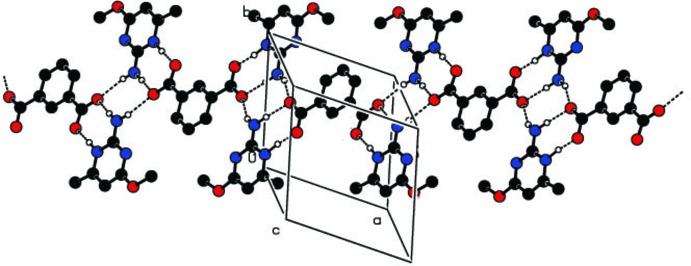
The *DDAA* array of quadruple hydrogen-bonding inter­actions with conjoined 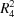
(8) and peripheral 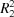
(8) ring motifs.

In the crystal, the protonated nitro­gen atoms (N1*A* and N1*B*) and the 2-amino group nitro­gen atoms (N2*A* and N2*B*) of the cations form two pairs of N—H⋯O hydrogen bonds with carboxyl O-atom acceptors (O3, O5) and (O2, O4), respectively, of the isophalate anion (Table 1 ▸ and Fig. 1 ▸). These form eight-membered ring motifs with graph-set notation 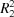
(8) on either side of the pyrimidine dianion. The ring units are cyclically linked across a crystalligraphic inversion centre through four N—H⋯O hydrogen bonds [graph set 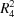
(8)], providing a *DDAA* array of quadruple hydrogen bonds (*D* = H-atom donor, *A* = H-atom acceptor) represented by the overall graph-set notation 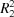
(8), 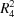
(8), 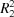
(8), as shown in Fig. 2 ▸. The same type of conjoined motif has been reported in the crystal structures of trimethoprim hydrogen glutarate (Robert *et al.*, 2001 ▸), 2-amino-4-meth­oxy-6-methyl­pyridinium tri­fluoro­acetate (Jeevaraj *et al.*, 2016 ▸) and 2-amino-4-meth­oxy-6-methyl­pyrimidinium 2-hy­droxy­benzoate (Jeevaraj *et al.*, 2017 ▸).

**Figure 3:**
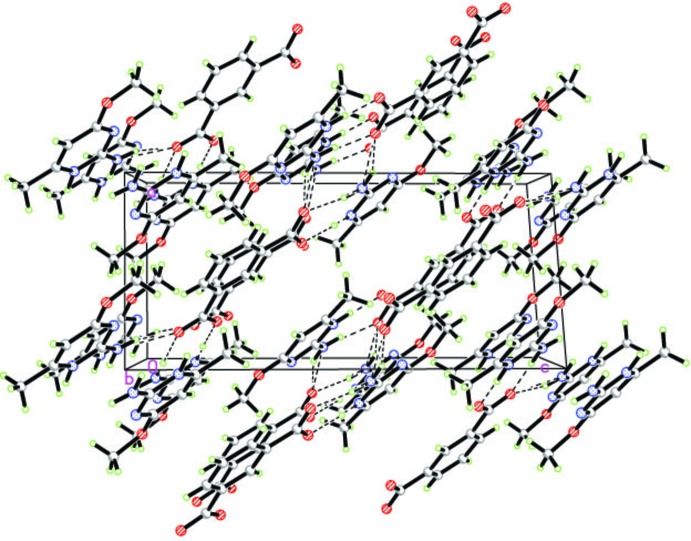
Crystal packing of the title compound in the unit cell viewed along *b*, with hydrogen bonds shown as dashed lines.

The extension of the crystal structure is through lateral duplex N2*A*— H⋯O2^ii^ and N2*B*—H⋯O4^i^ hydrogen bonds in centrosymmetric 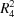
(8) inter­actions (for symmetry codes, see Table 1 ▸). These inter­actions result in one-dimensional ribbon structures extending along [211] (Fig. 3 ▸). The crystal structure is further stablized by π–π stacking inter­actions between the aromatic rings of the pyrimidine cations, having centroid–centroid separations *Cg*⋯*Cg* ^iii^ of 3.6337 (9) for cation *B* and *Cg*⋯*Cg* ^iv^ of 3.7260 (9) Å for cation *A* [symmetry codes: (iii) −*x* + 2, −*y* + 1, −*z* + 1; (iv) −*x*, −*y*, −*z*].

## Hirshfeld surface analysis   

**Figure 4:**
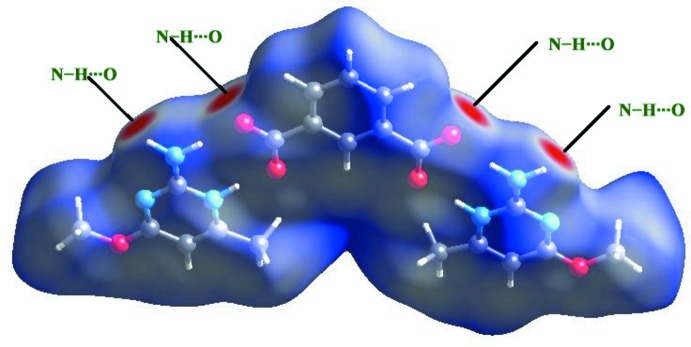
The three-dimensional *d*_norm_ surface of the salt.

**Figure 5:**
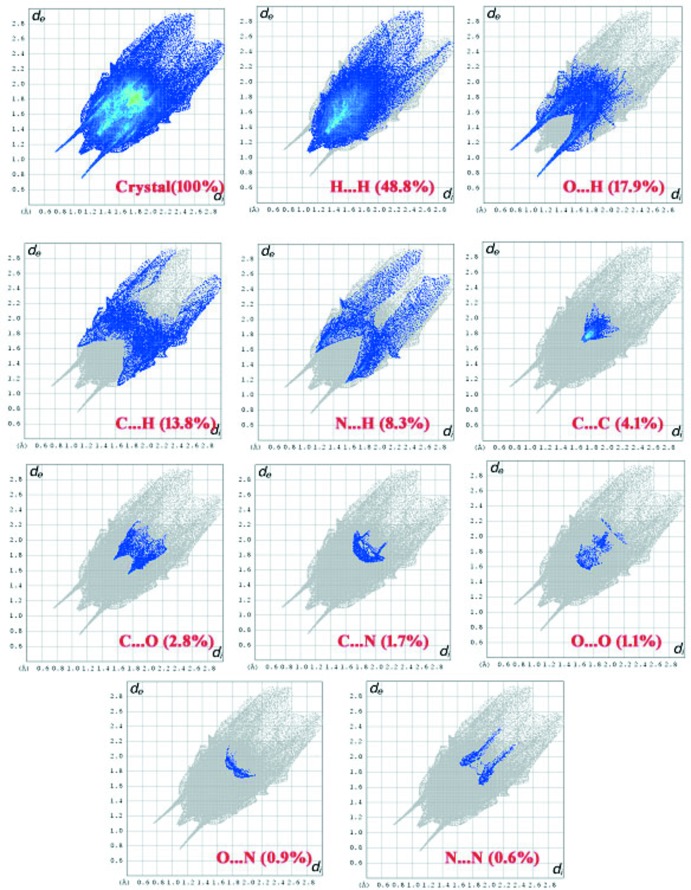
Two-dimensional fingerprint plots of the crystal and relative contribution of the atom pairs to the Hirshfeld surface.

The *d*_norm_ parameter takes negative or positive values depending upon whether the inter­molecular close contact is shorter or longer than the van der Waals radii, respectively (Spackman & Jayatilaka, 2009 ▸; McKinnon *et al.*, 2007 ▸). The 3D *d*_norm_ surface of the title salt is shown in Fig. 4 ▸. Colours are used to illustrate the contribution of inter­molecular contacts present in the crystal structure with red inidicating N—H⋯O inter­actions. Two-dimensional fingerprint images are depicted in Fig. 5 ▸, and from this study it is revealed that the H⋯H inter­actions present (48.8%) are a major contributor whereas O⋯H/H⋯O (17.9%), C⋯H/H⋯C (13.8%), N⋯H/H⋯N (8.3%), C⋯C (4.1%), C⋯O/O⋯C (2.8%), C⋯N/N⋯C (1.7%), O⋯O (1.1%), O⋯N/N⋯O (0.9%) and N⋯N (0.6%), have significant contribution to the total surface.

## Database survey   

A search of the Cambridge Structural Database (Version 5.37, update February 2014; Groom *et al.*, 2016 ▸) for 2-amino-4-meth­oxy-6-methyl­pyrimidine yielded only seven structures: VAQSOW, VAQSUC, VAQSEM, VAQSIQ, VAQRUB and VAQSAI (Aakeröy *et al.*, 2003 ▸); NUQTOJ (Jasinski *et al.*, 2010 ▸).

## Synthesis and crystallization   

The title compound was synthesized in a reaction involving a hot methano­lic solution (20 ml) of 2-amino-4-meth­oxy-6-methyl­pyrimidine (139 mg, 1.0 mmol) and a hot methano­lic solution (20 ml) of isophthalic acid (166 mg, 1.0 mmol). The two solutions were mixed and stirred on a heating magnetic stirrer for few minutes. The colorless solution was cooled and kept at room temperature for slow evaporation. After a few days, the crystals of the title compound suitable for the X-ray analysis appeared, yield 65%.

## Refinement   

Crystal data, data collection and structure refinement details are summarized in Table 2 ▸. The hydrogen atoms were positioned geometrically (N—H = 0.86 Å and C—H = 0.96 or 0.93 Å) and were refined using a riding model with *U*_iso_(H) = 1.2 *U*_eq_(N or C) or 1.5*U*_eq_(methyl C).

## Supplementary Material

Crystal structure: contains datablock(s) global, I, 1. DOI: 10.1107/S2056989017013950/zs2389sup1.cif

Structure factors: contains datablock(s) I. DOI: 10.1107/S2056989017013950/zs2389Isup2.hkl

Supporting information file. DOI: 10.1107/S2056989017013950/zs2389Isup3.cml

CCDC reference: 1559277

Additional supporting information:  crystallographic information; 3D view; checkCIF report

## Figures and Tables

**Table 1:** Hydrogen-bond geometry (Å, °)

*D*—H⋯*A*	*D*—H	H⋯*A*	*D*⋯*A*	*D*—H⋯*A*
N2*B*—H2*B*1⋯O4^i^	0.86	2.04	2.8150 (19)	150
N1*A*—H1*A*⋯O3	0.86	1.74	2.5921 (17)	171
N1*B*—H1*B*⋯O5	0.86	1.79	2.6448 (17)	175
N2*B*—H2*B*2⋯O4	0.86	1.93	2.7648 (19)	164
N2*A*—H2*A*1⋯O2^ii^	0.86	2.03	2.805 (2)	150
N2*A*—H2*A*2⋯O2	0.86	1.95	2.803 (2)	172

Symmetry codes: (i) 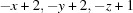; (ii) 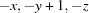.

**Table 2:** Experimental details

Crystal data	
Chemical formula	2C_6_H_10_N_3_O^+^·C_8_H_4_O_4_^2−^
*M*_r_	444.45
Crystal system, space group	Triclinic, *P* 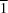
Temperature (K)	296
*a*, *b*, *c* (Å)	8.1346 (3), 8.2092 (3), 17.2340 (6)
α, β, γ (°)	92.4728 (12), 91.3245 (13), 107.0413 (12)
*V* (Å^3^)	1098.54 (7)
*Z*	2
Radiation type	Mo *K*α
μ (mm^−1^)	0.10
Crystal size (mm)	0.62 × 0.42 × 0.35

Data collection	
Diffractometer	Bruker Kappa APEXII CCD
Absorption correction	Multi-scan (*SADABS*; Bruker, 2004 ▸)
*T*_min_, *T*_max_	0.893, 0.920
No. of measured, independent and observed [*I* > 2σ(*I*)] reflections	36645, 5061, 3717
*R*_int_	0.028
(sin θ/λ)_max_ (Å^−1^)	0.650

Refinement	
*R*[*F* ^2^ > 2σ(*F* ^2^)], *wR*(*F* ^2^), *S*	0.047, 0.151, 1.09
No. of reflections	5060
No. of parameters	293
H-atom treatment	H-atom parameters constrained
Δρ_max_, Δρ_min_ (e Å^−3^)	0.21, −0.19

Computer programs: *APEX2*, *XPREP* and *SAINT* (Bruker, 2002), *SHELXS97* and *SHELXTL* (Sheldrick, 2008 ▸), *SHELXL2015* (Sheldrick, 2015 ▸) and *PLATON* (Spek, 2009 ▸).

## supplementary crystallographic information

### Crystal data

**Table:**

2C_6_H_10_N_3_O^+^·C_8_H_4_O_4_^2^^−^	*Z* = 2
*M**_r_* = 444.45	*F*(000) = 468
Triclinic, *P*1	*D*_x_ = 1.344 Mg m^−^^3^
*a* = 8.1346 (3) Å	Mo *K*α radiation, λ = 0.71073 Å
*b* = 8.2092 (3) Å	Cell parameters from 5061 reflections
*c* = 17.2340 (6) Å	θ = 2.4–27.5°
α = 92.4728 (12)°	µ = 0.10 mm^−^^1^
β = 91.3245 (13)°	*T* = 296 K
γ = 107.0413 (12)°	Block, colourless
*V* = 1098.54 (7) Å^3^	0.62 × 0.42 × 0.35 mm

### Data collection

**Table:**

Bruker Kappa APEXII CCD diffractometer	3717 reflections with *I* > 2σ(*I*)
Radiation source: fine-focus sealed tube	*R*_int_ = 0.028
ω and φ scans	θ_max_ = 27.5°, θ_min_ = 2.4°
Absorption correction: multi-scan (SADABS; Bruker, 2004)	*h* = −10→10
*T*_min_ = 0.893, *T*_max_ = 0.920	*k* = −10→10
36645 measured reflections	*l* = −22→22
5061 independent reflections	

### Refinement

**Table:**

Refinement on *F*^2^	0 restraints
Least-squares matrix: full	Hydrogen site location: inferred from neighbouring sites
*R*[*F*^2^ > 2σ(*F*^2^)] = 0.047	H-atom parameters constrained
*wR*(*F*^2^) = 0.151	*w* = 1/[σ^2^(*F*_o_^2^) + (0.0712*P*)^2^ + 0.2201*P*] where *P* = (*F*_o_^2^ + 2*F*_c_^2^)/3
*S* = 1.09	(Δ/σ)_max_ = 0.009
5060 reflections	Δρ_max_ = 0.21 e Å^−^^3^
293 parameters	Δρ_min_ = −0.19 e Å^−^^3^

### Special details

**Table:**

Geometry. All esds (except the esd in the dihedral angle between two l.s. planes) are estimated using the full covariance matrix. The cell esds are taken into account individually in the estimation of esds in distances, angles and torsion angles; correlations between esds in cell parameters are only used when they are defined by crystal symmetry. An approximate (isotropic) treatment of cell esds is used for estimating esds involving l.s. planes.

### Fractional atomic coordinates and isotropic or equivalent isotropic displacement parameters (Å^2^)

**Table:**

	*x*	*y*	*z*	*U*_iso_*/*U*_eq_	
N1A	−0.01236 (16)	0.13584 (16)	0.12598 (7)	0.0429 (3)	
H1A	0.055899	0.227779	0.147811	0.051*	
N1B	0.79527 (16)	0.53363 (16)	0.52792 (7)	0.0432 (3)	
H1B	0.740866	0.559370	0.489182	0.052*	
N2B	0.92978 (19)	0.81823 (16)	0.55416 (8)	0.0538 (4)	
H2B1	1.001350	0.900179	0.581201	0.065*	
H2B2	0.872417	0.840238	0.515499	0.065*	
N2A	−0.0872 (2)	0.29572 (17)	0.03446 (8)	0.0598 (4)	
H2A1	−0.146357	0.305927	−0.006085	0.072*	
H2A2	−0.015998	0.384548	0.057300	0.072*	
N3B	0.99869 (17)	0.62724 (16)	0.63248 (7)	0.0429 (3)	
N3A	−0.21746 (17)	0.00644 (16)	0.02535 (8)	0.0467 (3)	
O1B	1.05403 (17)	0.42661 (15)	0.70799 (7)	0.0586 (3)	
O1A	−0.33778 (18)	−0.28258 (16)	0.02360 (9)	0.0717 (4)	
O2	0.16941 (18)	0.57355 (16)	0.10408 (8)	0.0695 (4)	
O3	0.21834 (16)	0.39923 (14)	0.18953 (7)	0.0589 (3)	
O4	0.79842 (17)	0.88112 (15)	0.41293 (7)	0.0643 (4)	
O5	0.64086 (15)	0.61013 (14)	0.40389 (7)	0.0555 (3)	
C1B	0.90804 (19)	0.65930 (18)	0.57191 (8)	0.0410 (3)	
C1A	−0.10574 (19)	0.14466 (19)	0.06145 (9)	0.0425 (3)	
C2B	0.7665 (2)	0.36687 (19)	0.54402 (9)	0.0456 (4)	
C2A	−0.0249 (2)	−0.0166 (2)	0.15691 (10)	0.0487 (4)	
C3B	0.8530 (2)	0.3293 (2)	0.60541 (10)	0.0515 (4)	
H3BA	0.836264	0.217138	0.618682	0.062*	
C3A	−0.1312 (3)	−0.1589 (2)	0.12097 (12)	0.0605 (5)	
H3AA	−0.139979	−0.266164	0.139045	0.073*	
C4B	0.9690 (2)	0.4660 (2)	0.64837 (9)	0.0454 (4)	
C4A	−0.2281 (2)	−0.1402 (2)	0.05555 (10)	0.0517 (4)	
C5B	0.6421 (2)	0.2386 (2)	0.49067 (12)	0.0607 (5)	
H5BA	0.532723	0.261138	0.490466	0.091*	
H5BB	0.628885	0.126229	0.508184	0.091*	
H5BC	0.684565	0.246030	0.439034	0.091*	
C5A	0.0783 (3)	−0.0105 (3)	0.23016 (12)	0.0660 (5)	
H5AA	0.193899	0.060400	0.224298	0.099*	
H5AB	0.079853	−0.123711	0.241133	0.099*	
H5AC	0.027678	0.035789	0.272238	0.099*	
C6B	1.1837 (3)	0.5631 (2)	0.74878 (11)	0.0634 (5)	
H6BA	1.237028	0.517871	0.789506	0.095*	
H6BB	1.131620	0.644482	0.770828	0.095*	
H6BC	1.269111	0.618162	0.713223	0.095*	
C6A	−0.4472 (3)	−0.2673 (3)	−0.04086 (14)	0.0779 (6)	
H6AA	−0.527231	−0.376790	−0.054897	0.117*	
H6AB	−0.378123	−0.226479	−0.084393	0.117*	
H6AC	−0.509236	−0.188282	−0.026269	0.117*	
C7	0.2483 (2)	0.5433 (2)	0.16146 (9)	0.0436 (4)	
C8	0.38688 (18)	0.68720 (19)	0.20172 (8)	0.0389 (3)	
C9	0.4277 (2)	0.8500 (2)	0.17444 (9)	0.0472 (4)	
H9A	0.372680	0.869938	0.129514	0.057*	
C10	0.5503 (2)	0.9830 (2)	0.21387 (10)	0.0553 (4)	
H10A	0.577106	1.092201	0.195488	0.066*	
C11	0.6331 (2)	0.9540 (2)	0.28053 (10)	0.0476 (4)	
H11A	0.714413	1.044147	0.307115	0.057*	
C12	0.59580 (18)	0.79158 (19)	0.30793 (8)	0.0391 (3)	
C13	0.47164 (18)	0.65826 (19)	0.26823 (8)	0.0389 (3)	
H13A	0.445286	0.548907	0.286444	0.047*	
C14	0.68577 (19)	0.7582 (2)	0.38013 (9)	0.0431 (3)	

### Atomic displacement parameters (Å^2^)

**Table:**

	*U*^11^	*U*^22^	*U*^33^	*U*^12^	*U*^13^	*U*^23^
N1A	0.0455 (7)	0.0362 (7)	0.0437 (7)	0.0074 (5)	−0.0061 (5)	0.0030 (5)
N1B	0.0458 (7)	0.0367 (7)	0.0409 (7)	0.0029 (5)	−0.0040 (5)	0.0018 (5)
N2B	0.0680 (9)	0.0341 (7)	0.0506 (8)	0.0035 (6)	−0.0239 (7)	0.0028 (6)
N2A	0.0728 (10)	0.0390 (7)	0.0543 (8)	−0.0031 (7)	−0.0290 (7)	0.0093 (6)
N3B	0.0522 (7)	0.0362 (7)	0.0371 (6)	0.0081 (5)	−0.0031 (5)	0.0036 (5)
N3A	0.0479 (7)	0.0383 (7)	0.0468 (7)	0.0024 (6)	−0.0024 (6)	−0.0007 (6)
O1B	0.0770 (8)	0.0444 (7)	0.0530 (7)	0.0152 (6)	−0.0096 (6)	0.0127 (5)
O1A	0.0745 (9)	0.0378 (7)	0.0886 (10)	−0.0031 (6)	−0.0095 (8)	−0.0065 (6)
O2	0.0835 (9)	0.0479 (7)	0.0617 (8)	−0.0022 (6)	−0.0408 (7)	0.0097 (6)
O3	0.0671 (8)	0.0410 (6)	0.0575 (7)	0.0003 (5)	−0.0270 (6)	0.0067 (5)
O4	0.0736 (8)	0.0427 (7)	0.0608 (7)	−0.0046 (6)	−0.0352 (6)	0.0032 (5)
O5	0.0626 (7)	0.0425 (6)	0.0511 (7)	0.0008 (5)	−0.0204 (5)	0.0053 (5)
C1B	0.0473 (8)	0.0342 (7)	0.0371 (7)	0.0055 (6)	−0.0010 (6)	0.0008 (6)
C1A	0.0447 (8)	0.0376 (8)	0.0408 (8)	0.0055 (6)	−0.0031 (6)	0.0015 (6)
C2B	0.0465 (8)	0.0344 (8)	0.0488 (9)	0.0005 (6)	0.0089 (7)	0.0004 (6)
C2A	0.0509 (9)	0.0431 (9)	0.0538 (9)	0.0152 (7)	0.0031 (7)	0.0114 (7)
C3B	0.0630 (10)	0.0332 (8)	0.0540 (9)	0.0064 (7)	0.0052 (8)	0.0082 (7)
C3A	0.0695 (11)	0.0369 (9)	0.0731 (12)	0.0117 (8)	−0.0006 (9)	0.0108 (8)
C4B	0.0552 (9)	0.0393 (8)	0.0408 (8)	0.0112 (7)	0.0056 (7)	0.0078 (6)
C4A	0.0518 (9)	0.0378 (8)	0.0591 (10)	0.0038 (7)	0.0044 (8)	−0.0025 (7)
C5B	0.0604 (10)	0.0429 (9)	0.0657 (11)	−0.0034 (8)	−0.0003 (9)	−0.0058 (8)
C5A	0.0691 (12)	0.0608 (11)	0.0697 (12)	0.0200 (9)	−0.0108 (10)	0.0205 (10)
C6B	0.0791 (12)	0.0531 (10)	0.0561 (10)	0.0177 (9)	−0.0205 (9)	0.0053 (8)
C6A	0.0690 (12)	0.0590 (12)	0.0871 (15)	−0.0055 (10)	−0.0173 (11)	−0.0166 (11)
C7	0.0466 (8)	0.0404 (8)	0.0394 (8)	0.0073 (6)	−0.0112 (6)	0.0004 (6)
C8	0.0378 (7)	0.0383 (8)	0.0379 (7)	0.0076 (6)	−0.0033 (6)	−0.0007 (6)
C9	0.0502 (8)	0.0441 (8)	0.0442 (8)	0.0097 (7)	−0.0116 (7)	0.0046 (7)
C10	0.0610 (10)	0.0372 (8)	0.0610 (10)	0.0041 (7)	−0.0126 (8)	0.0094 (7)
C11	0.0473 (8)	0.0369 (8)	0.0515 (9)	0.0029 (6)	−0.0118 (7)	−0.0022 (7)
C12	0.0366 (7)	0.0401 (8)	0.0384 (7)	0.0086 (6)	−0.0031 (6)	−0.0008 (6)
C13	0.0389 (7)	0.0357 (7)	0.0387 (7)	0.0065 (6)	−0.0051 (6)	0.0003 (6)
C14	0.0425 (8)	0.0407 (8)	0.0412 (8)	0.0062 (6)	−0.0088 (6)	−0.0018 (6)

### Geometric parameters (Å, º)

**Table:**

N1A—C1A	1.3488 (19)	C3B—C4B	1.405 (2)
N1A—C2A	1.359 (2)	C3B—H3BA	0.9300
N1A—H1A	0.8600	C3A—C4A	1.400 (3)
N1B—C1B	1.3514 (19)	C3A—H3AA	0.9300
N1B—C2B	1.361 (2)	C5B—H5BA	0.9600
N1B—H1B	0.8600	C5B—H5BB	0.9600
N2B—C1B	1.3149 (19)	C5B—H5BC	0.9600
N2B—H2B1	0.8600	C5A—H5AA	0.9600
N2B—H2B2	0.8600	C5A—H5AB	0.9600
N2A—C1A	1.312 (2)	C5A—H5AC	0.9600
N2A—H2A1	0.8600	C6B—H6BA	0.9600
N2A—H2A2	0.8600	C6B—H6BB	0.9600
N3B—C4B	1.3166 (19)	C6B—H6BC	0.9600
N3B—C1B	1.3433 (19)	C6A—H6AA	0.9600
N3A—C4A	1.312 (2)	C6A—H6AB	0.9600
N3A—C1A	1.3450 (19)	C6A—H6AC	0.9600
O1B—C4B	1.3284 (19)	C7—C8	1.503 (2)
O1B—C6B	1.438 (2)	C8—C9	1.385 (2)
O1A—C4A	1.333 (2)	C8—C13	1.389 (2)
O1A—C6A	1.440 (3)	C9—C10	1.384 (2)
O2—C7	1.2394 (18)	C9—H9A	0.9300
O3—C7	1.2565 (19)	C10—C11	1.383 (2)
O4—C14	1.2500 (18)	C10—H10A	0.9300
O5—C14	1.2522 (19)	C11—C12	1.384 (2)
C2B—C3B	1.353 (2)	C11—H11A	0.9300
C2B—C5B	1.492 (2)	C12—C13	1.3934 (19)
C2A—C3A	1.348 (3)	C12—C14	1.505 (2)
C2A—C5A	1.491 (2)	C13—H13A	0.9300
			
C1A—N1A—C2A	120.76 (13)	H5BA—C5B—H5BC	109.5
C1A—N1A—H1A	119.6	H5BB—C5B—H5BC	109.5
C2A—N1A—H1A	119.6	C2A—C5A—H5AA	109.5
C1B—N1B—C2B	120.99 (14)	C2A—C5A—H5AB	109.5
C1B—N1B—H1B	119.5	H5AA—C5A—H5AB	109.5
C2B—N1B—H1B	119.5	C2A—C5A—H5AC	109.5
C1B—N2B—H2B1	120.0	H5AA—C5A—H5AC	109.5
C1B—N2B—H2B2	120.0	H5AB—C5A—H5AC	109.5
H2B1—N2B—H2B2	120.0	O1B—C6B—H6BA	109.5
C1A—N2A—H2A1	120.0	O1B—C6B—H6BB	109.5
C1A—N2A—H2A2	120.0	H6BA—C6B—H6BB	109.5
H2A1—N2A—H2A2	120.0	O1B—C6B—H6BC	109.5
C4B—N3B—C1B	116.45 (13)	H6BA—C6B—H6BC	109.5
C4A—N3A—C1A	116.01 (14)	H6BB—C6B—H6BC	109.5
C4B—O1B—C6B	117.64 (13)	O1A—C6A—H6AA	109.5
C4A—O1A—C6A	117.94 (15)	O1A—C6A—H6AB	109.5
N2B—C1B—N3B	119.21 (13)	H6AA—C6A—H6AB	109.5
N2B—C1B—N1B	118.44 (14)	O1A—C6A—H6AC	109.5
N3B—C1B—N1B	122.34 (14)	H6AA—C6A—H6AC	109.5
N2A—C1A—N3A	119.59 (14)	H6AB—C6A—H6AC	109.5
N2A—C1A—N1A	117.70 (13)	O2—C7—O3	124.16 (14)
N3A—C1A—N1A	122.70 (14)	O2—C7—C8	118.79 (14)
C3B—C2B—N1B	118.51 (14)	O3—C7—C8	117.04 (13)
C3B—C2B—C5B	125.04 (15)	C9—C8—C13	119.47 (13)
N1B—C2B—C5B	116.44 (15)	C9—C8—C7	120.64 (13)
C3A—C2A—N1A	118.38 (16)	C13—C8—C7	119.86 (13)
C3A—C2A—C5A	125.42 (16)	C10—C9—C8	120.16 (14)
N1A—C2A—C5A	116.17 (15)	C10—C9—H9A	119.9
C2B—C3B—C4B	117.56 (15)	C8—C9—H9A	119.9
C2B—C3B—H3BA	121.2	C11—C10—C9	120.19 (15)
C4B—C3B—H3BA	121.2	C11—C10—H10A	119.9
C2A—C3A—C4A	117.88 (16)	C9—C10—H10A	119.9
C2A—C3A—H3AA	121.1	C10—C11—C12	120.42 (14)
C4A—C3A—H3AA	121.1	C10—C11—H11A	119.8
N3B—C4B—O1B	119.14 (14)	C12—C11—H11A	119.8
N3B—C4B—C3B	124.12 (15)	C11—C12—C13	119.16 (13)
O1B—C4B—C3B	116.73 (14)	C11—C12—C14	120.92 (13)
N3A—C4A—O1A	119.48 (17)	C13—C12—C14	119.91 (13)
N3A—C4A—C3A	124.20 (15)	C8—C13—C12	120.59 (14)
O1A—C4A—C3A	116.31 (16)	C8—C13—H13A	119.7
C2B—C5B—H5BA	109.5	C12—C13—H13A	119.7
C2B—C5B—H5BB	109.5	O4—C14—O5	124.55 (14)
H5BA—C5B—H5BB	109.5	O4—C14—C12	117.59 (14)
C2B—C5B—H5BC	109.5	O5—C14—C12	117.85 (13)
			
C4B—N3B—C1B—N2B	178.73 (15)	C1A—N3A—C4A—C3A	−0.4 (3)
C4B—N3B—C1B—N1B	−1.7 (2)	C6A—O1A—C4A—N3A	−3.3 (3)
C2B—N1B—C1B—N2B	−179.43 (14)	C6A—O1A—C4A—C3A	176.28 (18)
C2B—N1B—C1B—N3B	1.0 (2)	C2A—C3A—C4A—N3A	2.5 (3)
C4A—N3A—C1A—N2A	179.66 (16)	C2A—C3A—C4A—O1A	−176.97 (17)
C4A—N3A—C1A—N1A	−1.6 (2)	O2—C7—C8—C9	−1.1 (2)
C2A—N1A—C1A—N2A	−179.80 (15)	O3—C7—C8—C9	179.99 (15)
C2A—N1A—C1A—N3A	1.4 (2)	O2—C7—C8—C13	177.05 (15)
C1B—N1B—C2B—C3B	0.2 (2)	O3—C7—C8—C13	−1.9 (2)
C1B—N1B—C2B—C5B	−178.77 (14)	C13—C8—C9—C10	−0.9 (2)
C1A—N1A—C2A—C3A	0.8 (2)	C7—C8—C9—C10	177.20 (15)
C1A—N1A—C2A—C5A	−177.42 (15)	C8—C9—C10—C11	0.3 (3)
N1B—C2B—C3B—C4B	−0.6 (2)	C9—C10—C11—C12	0.8 (3)
C5B—C2B—C3B—C4B	178.30 (16)	C10—C11—C12—C13	−1.1 (2)
N1A—C2A—C3A—C4A	−2.6 (3)	C10—C11—C12—C14	179.64 (15)
C5A—C2A—C3A—C4A	175.43 (18)	C9—C8—C13—C12	0.6 (2)
C1B—N3B—C4B—O1B	−179.52 (14)	C7—C8—C13—C12	−177.54 (14)
C1B—N3B—C4B—C3B	1.3 (2)	C11—C12—C13—C8	0.4 (2)
C6B—O1B—C4B—N3B	−4.3 (2)	C14—C12—C13—C8	179.69 (14)
C6B—O1B—C4B—C3B	174.96 (16)	C11—C12—C14—O4	−2.0 (2)
C2B—C3B—C4B—N3B	−0.1 (3)	C13—C12—C14—O4	178.71 (15)
C2B—C3B—C4B—O1B	−179.39 (15)	C11—C12—C14—O5	176.88 (15)
C1A—N3A—C4A—O1A	179.08 (15)	C13—C12—C14—O5	−2.4 (2)

### Hydrogen-bond geometry (Å, º)

**Table:**

*D*—H···*A*	*D*—H	H···*A*	*D*···*A*	*D*—H···*A*
N2*B*—H2*B*1···O4^i^	0.86	2.04	2.8150 (19)	150
N1*A*—H1*A*···O3	0.86	1.74	2.5921 (17)	171
N1*B*—H1*B*···O5	0.86	1.79	2.6448 (17)	175
N2*B*—H2*B*2···O4	0.86	1.93	2.7648 (19)	164
N2*A*—H2*A*1···O2^ii^	0.86	2.03	2.805 (2)	150
N2*A*—H2*A*2···O2	0.86	1.95	2.803 (2)	172

Symmetry codes: (i) −*x*+2, −*y*+2, −*z*+1; (ii) −*x*, −*y*+1, −*z*.
